# Financial incentives for objectively-measured physical activity or weight loss in adults with chronic health conditions: A meta-analysis

**DOI:** 10.1371/journal.pone.0203939

**Published:** 2018-09-25

**Authors:** Yusi Gong, Taylor P. Trentadue, Swastina Shrestha, Elena Losina, Jamie E. Collins

**Affiliations:** 1 Orthopaedic and Arthritis Center for Outcomes Research (OrACORe) and the Policy and Innovation eValuation in Orthopaedic Treatments (PIVOT) Center, Department of Orthopaedic Surgery, Brigham and Women’s Hospital, Boston, Massachusetts, United States of America; 2 Department of Orthopedics, Harvard Medical School, Boston, Massachusetts, United States of America; 3 Section of Clinical Sciences, Division of Rheumatology, Immunology and Allergy, Brigham and Women’s Hospital, Boston, Massachusetts, United States of America; 4 Department of Biostatistics, Boston University School of Public Health, Boston, Massachusetts, United States of America; Universita degli Studi di Ferrara, ITALY

## Abstract

**Objective:**

We conducted a meta-analysis and systematic review of published randomized controlled trials (RCTs) to evaluate the impact of financial incentives (FI) on objectively-measured physical activity (PA) and weight loss (WL) in adults with sedentary behavior or chronic health conditions.

**Evidence review:**

We performed a systematic search for RCTs published in English indexed in PubMed, Embase, or Web of Science through July 27, 2017. We limited our search to RCTs that involved an FI intervention with a monetary component, objectively-measured PA or WL outcomes, samples with either sedentary lifestyles or chronic health conditions, and a comparator group that did not receive performance-contingent FI. We calculated the mean difference and standardized mean difference (SMD) for each study and used a random effects model to summarize intervention efficacy. We used the Jadad scoring tool to assess the quality of the identified articles.

**Results:**

We abstracted data from 11 RCTs. Two of the 11 included studies focused on PA, totaling 126 intervention and 116 control subjects. Nine RCTs evaluated the effect of FI on WL, totaling 1,799 intervention and 1,483 control subjects. The combined estimate for change in daily steps was 940 (95%CI [306–1,574]) more in PA intervention groups than in control groups and 2.36 (95%CI [1.80–2.93]) more kilograms lost by WL intervention groups compared to control groups. The overall estimated SMD for both outcomes combined was 0.395 (95%CI [0.243–0.546; p<0.001]), favoring FI interventions.

**Conclusion:**

FI interventions are efficacious in increasing PA and WL in adults with chronic conditions or sedentary adults. Public health programs to increase PA or prevent chronic disease should consider incorporating FI to improve outcomes.

## Introduction

The prevalence of chronic health conditions and sedentary behavior is rising: over a third of US adults are obese,[1, 2] and between 5% and 13% of adults meet the recommended physical activity (PA) guidelines.[3–5] Limited PA and chronic conditions reduce quality of life,[6, 7] are predictors of increased morbidity and mortality,[8, 9] and are costly.[10, 11] PA and weight loss (WL) are associated with reduced risk of comorbidity incidence and progression.[12–14] This highlights the need for interventions to increase PA and promote WL.

When making decisions pertaining to long-term health, people disproportionately choose immediate gratification from unhealthy activities over rewards with delayed and uncertain outcomes, such as reduced disease risk.[15–19] Delay discounting is a risk factor for physical inactivity and obesity.[20, 21] Financial incentive (FI) interventions draw from behavioral economic theory, which aims to understand economic principles in the context of psychological and social influences.[15, 22] FI interventions provide immediate rewards contingent on engaging in healthful behaviors and offer extrinsic motivation for developing healthy habits.[23]

FI have been used in studies to promote behaviors such as smoking cessation, medication adherence, PA, and WL.[23–28] Successful FI interventions incorporate actuarial amounts, or the raw monetary values, of incentives that are valued high enough to be motivating.[29] Intervention duration is another important parameter in determining habit formation and successful achievement of behavioral goals. For many adults, successful habit formation develops over at least two months of engaging in the behavior.[30] Further, interventions that employ principles of loss aversion, the notion that the magnitude in dissatisfaction associated with losing acquisitions is greater than the satisfaction associated with actuarial equivalent gains, are often more successful than those that utilize gain incentive schemes.[17, 31]

Previous systematic reviews have evaluated the effects of FI for PA or WL,[23, 24, 26, 31, 32] but these reviews have included self-reported data, which may overestimate outcomes.[33] Further, those with chronic conditions may have different health behaviors and perceptions compared to an ostensibly healthy population,[34] which may result in different responses to financial incentives for PA or WL. We aim to use evidence from randomized controlled trials (RCTs) to determine the efficacy of FI for objectively-measured PA and WL in persons with chronic conditions or sedentary lifestyles and determine whether specific components of intervention design make FI interventions more efficacious.

## Methods

This study follows the Preferred Reporting Items for Systematic Reviews and Meta-Analyses (PRISMA) protocol[35] and was registered in the PROSPERO International Prospective Register of Systematic Reviews (2017:CRD42017058063).

### Identification of studies

We performed a systematic search of PubMed, Embase, and Web of Science for peer-reviewed articles published from database inception through July 27, 2017 that were available in English.[36] Included studies met search criteria in three categories: (1) FI or behavioral economics, (2) PA or WL outcomes, and (3) presence of a chronic condition or sedentary lifestyle. The search strings used in each database are provided in the Supporting Information (Appendix in S1 File).

### Inclusion and exclusion criteria

Two reviewers (YG and TPT) screened the titles and abstracts of the studies and excluded those failing to meet inclusion criteria. The inclusion criteria were: (1) subjects at least eighteen years of age; (2) RCT; (3) at least one intervention arm eligible for PA or WL performance-contingent FI independent of additional interventions; (4) sample had a chronic health condition or sedentary lifestyle; (5) study reported objective PA or WL measures at baseline and either the change in outcome or the outcome at follow-up; (6) intervention lasted at least 12 weeks; (7) intervention designed to promote PA or WL; and (8) study included a control arm that did not receive performance-contingent FI.

After the title and abstract screens, two reviewers (YG and TPT) independently reviewed the full text of articles to confirm that the studies met the inclusion criteria. The reviewers discussed and came to consensus on any discrepancies. The reviewers referenced the literature cited in each included study to identify any additional papers not captured with the original searches.

### Data abstraction

YG and TPT independently abstracted data from the eligible manuscripts and entered data in a Research Electronic Data Capture (REDCap) database.[37] We abstracted participant demographics and clinical characteristics; characteristics of the control arm; FI scheme, distribution, and amount; primary follow-up time point; and primary outcome data. For studies that included more than one FI or comparator arm, we abstracted data from all intervention arms.

YG and TPT compared the data abstracted from each study in REDCap, resolved discrepancies via discussion, and consulted two adjudicators (EL and JEC) to clarify study inclusion or data abstraction protocols. For each study, we calculated the maximum possible actuarial amounts of performance-contingent incentives that subjects could earn until the primary outcome timepoint. Foreign currencies were first converted to US dollars using IRS yearly average currency exchange rates.[38] We adjusted FI amounts to 2016 US dollars using the US Bureau of Labor Statistics Consumer Price Index.[39] We divided by study duration to calculate weekly maximum possible actuarial FI amounts to compare time-adjusted FI amounts between studies of different durations.

If data were not available in tables or within the text, we abstracted data from published figures using ImageJ.[40] We contacted authors via e-mail up to two times if numerical outcome data were not published (Table A in S1 File). If we were unable to obtain the data necessary to compute SMD, the study was excluded.

We evaluated the articles that met the inclusion criteria for quality using the Jadad assessment tool, a five-point scoring system that assesses RCTs based on appropriate methods of randomization, blinding, and withdrawal reporting.[41] Because of the nature of a FI intervention, participants and certain study staff could not be blinded to the arm assignment. Therefore, in accordance with guidelines, we evaluated whether data analysts and investigators were blinded to arm assignment in the studies.[42, 43]

### Statistical analysis

We abstracted the primary outcome—objectively-measured average daily steps or weight—from each study at baseline and at end-of-intervention follow-up. We calculated the mean difference, or the difference in change in outcome from baseline to follow-up between control and intervention groups, for each intervention arm. To standardize the results measured on disparate scales to a uniform outcome unit, we calculated the standardized mean difference (SMD) for each study.[44] The SMD is calculated by dividing the mean difference by the standard deviation (SD) of the outcome.[44] For a study that reported the SD of change in the intervention and control groups separately,[45] we pooled the SDs and used this as the denominator to divide the mean difference to create SMD. This study did not report the SD of change from baseline to final time point for each group,[45] so we imputed these values by calculating the correlation between baseline, final, and change SDs from studies that reported all three values.[46] We assumed that correlations derived from studies that reported SDs for all three values would apply to the remaining studies. Using the properties of variances, namely that the variance of the sum of two distributions is the sum of the variances of the distributions plus twice their covariance, we calculated the SD of change for the study that did not explicitly report it.[45]

In one study with a factorial design,[47] two arms received an FI intervention and two did not. For the intervention group, we report the outcome as the weighted mean of the primary outcomes from arms receiving the FI intervention. For the control group, we report the weighted mean of the primary outcome from the two arms that did not receive the FI intervention. The SDs for each group were pooled using the appropriate arms.

We assessed heterogeneity between the studies using the Cochrane’s Q, I^2^, and H statistics.[46, 48] We assessed the contribution of each study arm to the overall heterogeneity using the influence and the Q-term, which is the contribution of the study to Cochrane’s Q-statistic. We computed the influence for each study arm by comparing the overall pooled estimate with and without including the arm. In a sensitivity analysis, we excluded study arms with large contributions to heterogeneity based on these measures.[49]

We used a random effects model using maximum likelihood to calculate the final combined estimate of SMD across study arms overall and within each outcome type (PA and WL) separately. For studies that reported more than one intervention meeting our inclusion criteria, we included both arms and compared each arm to the control group. We included a random effect for study to account for the correlation between multiple arms coming from the same study and having the same comparator group, and split the sample size of the control group equally among intervention arms, as recommended by the Cochrane Handbook.[46] We used meta-regression to determine the contributions of study characteristics to treatment effect. Study characteristics included study setting (workplace vs. non-workplace); type of outcome (WL vs. PA); incentive characteristics: the financial incentive schema (deposit contract, lottery, loss incentive, gain incentive), weekly maximum possible actuarial amount of FI, intervention duration, and the incentive distribution schedule; control arm characteristics: frequency of counseling in the control arm and frequency that the study followed-up with the control arm about the primary outcome. First, each characteristic was evaluated separately in a univariate regression model in order to estimate stratified effect estiamtes for each level of the predictor variable. Then, we selected variables with p-values less than 0.05 from univariate meta-regression to include in the multivariable meta-regression. To control for potential differences in outcome depending on outcome type (WL vs. PA) we ran an additional meta-regression including the covariates selected from univariate meta-regression and outcome type.

## Results

### Studies identified

**Fig 1:**
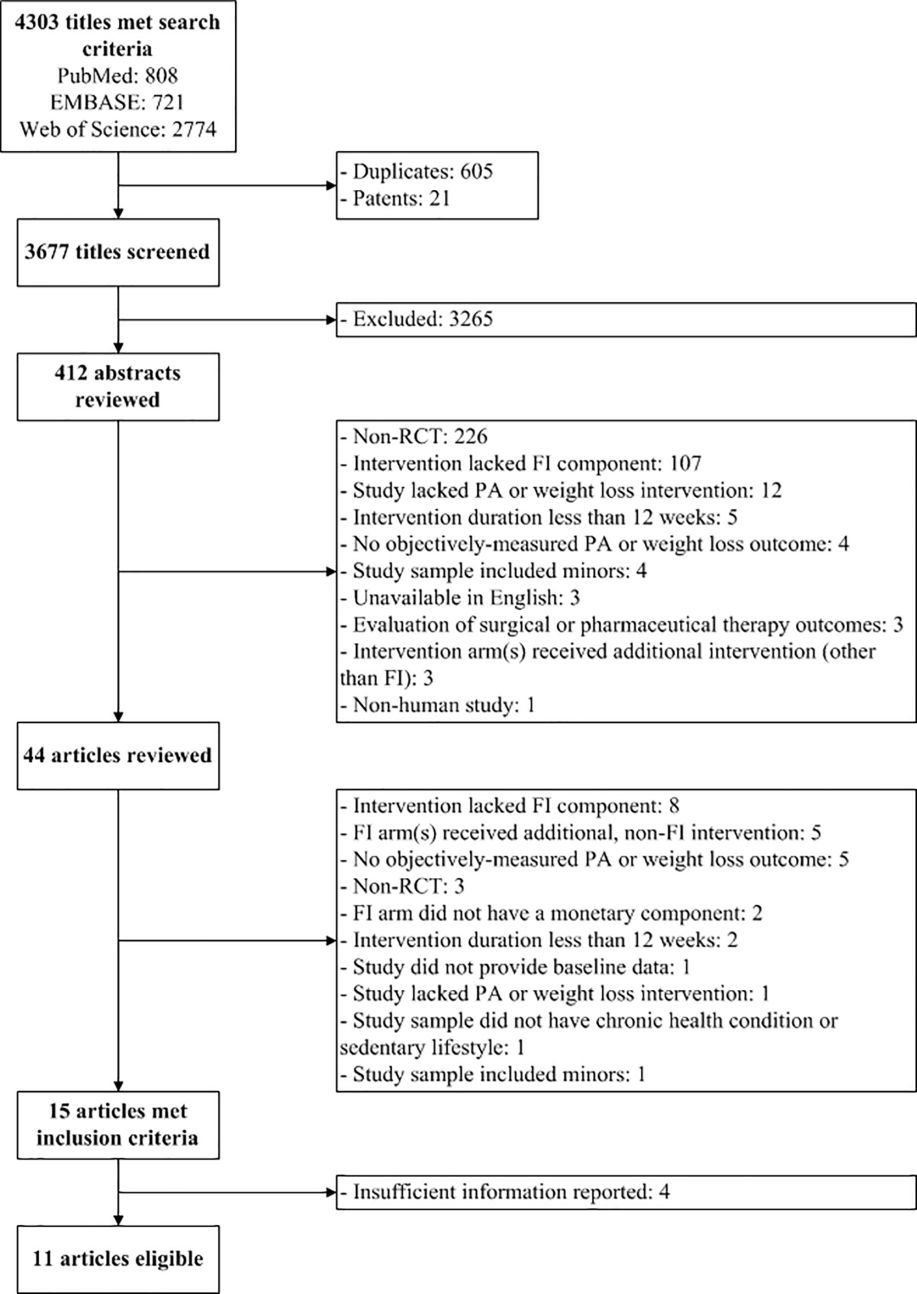
PRISMA flow consort diagram.[35].

A total of 4,303 citations met the search criteria, yielding 3,677 titles after removing duplicates and patents. From these, we identified 412 titles meeting search criteria and screened abstracts for inclusion criteria. Forty-four abstracts were eligible, which we considered in full-text review. Ultimately, 15 trials fulfilled all inclusion criteria. Four studies did not provide sufficient information to calculate SMD; thus, we included 11 papers containing 20 total intervention arms in the initial analysis. **Fig 1** presents the PRISMA flow diagram.[35]

### Heterogeneity assessment

**Fig 2:**
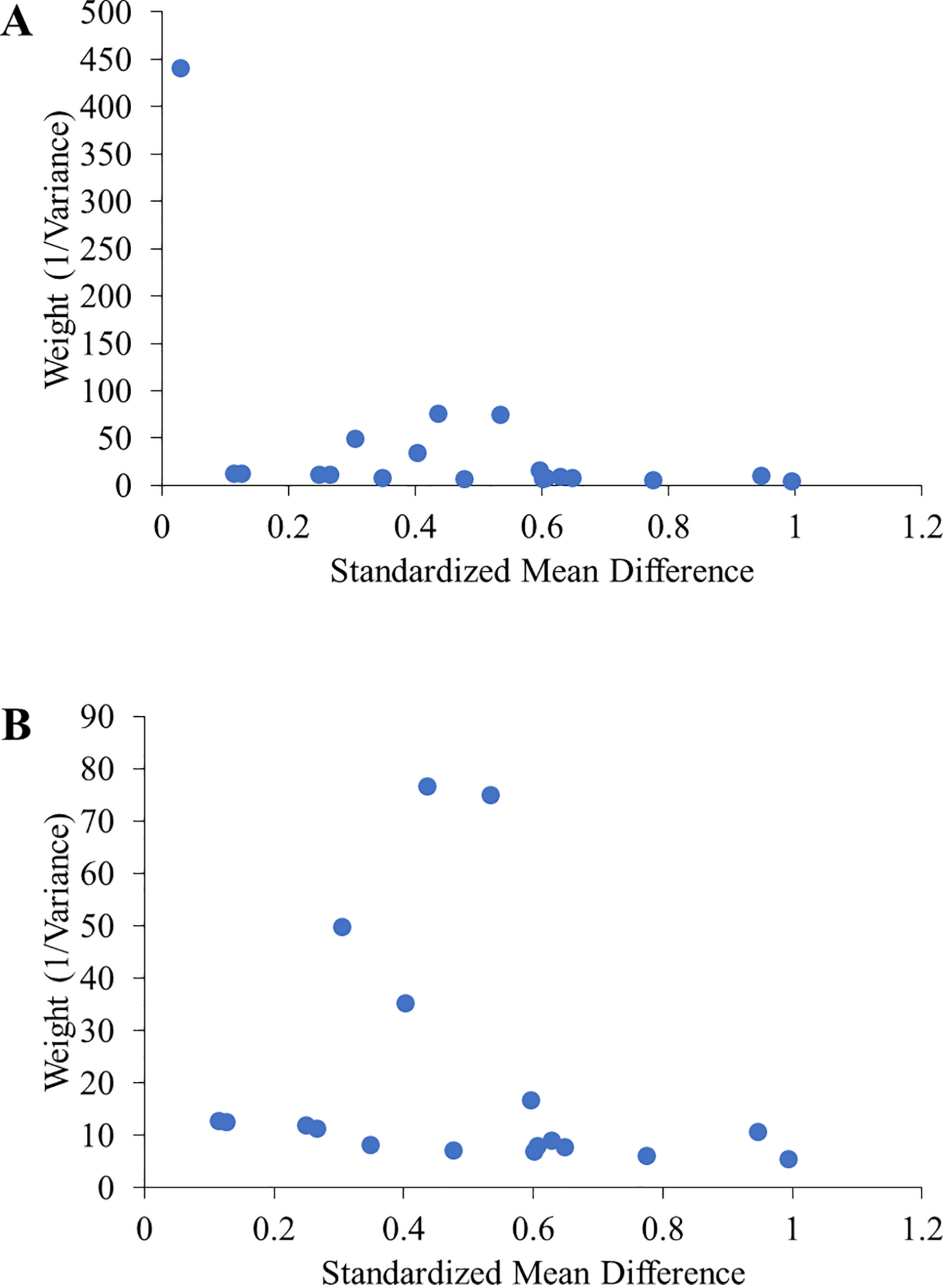
Funnel plot. (A) Funnel plot of all studies meeting inclusion criteria (n = 11 studies). Each point represents a study arm (n = 19 study arms). (B) Funnel plot with one outlier removed (n = 10 studies). Each point represents a study arm (n = 18 study arms).

Quantitative measures indicated considerable heterogeneity between the 11 studies (I^2^ = 0.62, 95%CI [0.38–0.77]; H = 1.63, 95%CI [1.27–2.09]). One study, Almeida (2015), had high weight (weight = 441.2, Q-term = 16.63), contributing substantially to heterogeneity.[45] In our sensitivity analysis, we excluded this study,[45] resulting in 10 included studies with 18 intervention arms. After removing Almeida (2015), heterogeneity was reduced considerably (I^2^ = -0.46, 95%CI [-1.92–0.27]; H = 0.83, 95%CI [0.59–1.17]). Publication bias was assessed graphically with funnel plots. The funnel plot without Almeida (2015) showed symmetry around the peak, failing to suggest publication bias. The funnel plots with and without the excluded studies are shown in **Fig 2**.

### Participant and trial characteristics

Among the 11 included studies, there were 11 control and **19** intervention arms for a total of 1,599 control and 1,925 intervention subjects, or 3,524 participants in total. In the two papers evaluating FI for PA,[47, 50] there were a total of 126 treatment subjects and 116 control subjects. Among the nine papers evaluating FI for WL,[45, 51–58] there were a total of 1,799 treatment subjects and 1,483 control subjects. The median sample size of all included studies was 132 participants (25^th^– 75^th^ percentile: 68–201.5) and ranged from 40 to 1790. Nine trials recruited overweight or obese subjects,[51–58] one recruited patients with sedentary lifestyles,[50] and one recruited subjects with osteoarthritis.[47] Four trials recruited participants from a workplace,[45, 53, 54, 56] three from hospitals,[47, 52, 58] two from the community,[50, 51] and one each from a rehabilitation clinic[55] and university.[57] **Table 1** describes study characteristics (see Table B in S1 File for a more comprehensive summary of study details).

**Table 1:** Study characteristics, baseline measures, and change in outcome measures.

			CONTROL			TREATMENT						
Study	Chronic condition	Site	N*	Baseline (SD)	Change (SD)	N	Baseline (SD)	Change (SD)	Mean age (SD)	Duration (weeks)	FI scheme	Maximum total expected payout (2016USD)
***Physical Activity (steps/day)***			*** ***	*** ***	*Increase in steps *	* *	* *	* Increase in steps *	*** ***	*** ***	*** ***	*** ***
**[50]**Harkins 2017	Sedentary lifestyle	Community	16	4851 (2500)	1624 (1133)	24	4794 (2826)	2769 (2108)	80 (6)	16	Gain incentive	$330
**[47]**Losina 2017	OA	Hospital	100	5578 (2984)	481 (2747)	102	5142 (3069)	1327 (2804)	65 (8)	24	Gain incentive	$305
***Weight Loss (kg)***		*** ***	* *	* *	*Weight loss *	* *	* *	*Weight loss *	* *	* *	* *	* *
**[51]**Finkelstein 2017	Obesity	Community	54	80.5 (13.1)	1.8 (3.7)	107	81.3 (12.1)	3.3 (3.7)	44 (10)	32	Gain incentive/Lottery	$385
**[52]**John 2011a	Obesity	Hospital	11	105.0 (10.9)	0.5 (6.3)	22	103.5 (10.6)	4.4 (6.2)	Not reported	32	Deposit contract	$1,645
**[52]**John 2011b	Obesity	Hospital	11	105.0 (10.9)	0.5 (6.3)	22	105.1 (14.5)	3.5 (5.8)	Not reported	32	Deposit contract	$1,645
**[53]**Kullgren 2013a	Obesity	Workplace	17	94.1 (14.0)	0.5 (5.1)	35	94.6 (12.0)	1.7 (4.8)	45 (10)	24	Loss incentive	$640
**[53]**Kullgren 2013b	Obesity	Workplace	18	94.1 (14.0)	0.5 (5.1)	35	98.0 (15.0)	4.8 (4.8)	45 (10)	24	Loss incentive	$640
**[54]**Kullgren 2016a	Obesity	Workplace	11	100.1 (15.6)	-0.5 (3.5)	33	100.7 (16.6)	2.0 (4.0)	44 (10)	24	Deposit contract	$538
**[54]**Kullgren 2016b	Obesity	Workplace	11	100.1 (15.6)	-0.5 (3.5)	33	97.1 (18.3)	2.4 (4.6)	44 (10)	24	Deposit contract	$1,076
**[54]**Kullgren 2016c	Obesity	Workplace	11	100.1 (15.6)	-0.5 (3.5)	33	108.2 (22.3)	1.0 (4.5)	44 (10)	24	Deposit contract	$1,613
**[55]**Paloyo 2015a	Obesity	Rehabilitation clinic	117	111.0 (20.0)	1.8 (4.9)	237	114.0 (23.0)	4.0 (5.2)	48 (9)	16	Gain incentive	$214
**[55]**Paloyo 2015b	Obesity	Rehabilitation clinic	117	111.0 (20.0)	1.8 (4.9)	229	114.0 (23.0)	4.9 (6.4)	48 (9)	16	Gain incentive	$428
**[56]**Patel 2016a	Obesity	Workplace	17	103.3 (18.5)	0.0 (6.5)	51	102.0 (16.2)	0.5 (5.5)	45 (10)	52	Gain incentive	$558
**[56]**Patel 2016b	Obesity	Workplace	17	103.3 (18.5)	0.0 (6.5)	50	103.7 (17.1)	0.6 (5.7)	45 (10)	52	Gain incentive	$558
**[56]**Patel 2016c	Obesity	Workplace	16	103.3 (18.5)	0.0 (6.5)	50	103.9 (17.2)	0.5 (1.0)	45 (10)	52	Lottery	$558
**[57]**Shin 2017	Obesity	University	35	91.6 (9.8)	1.1 (2.9)	35	92.7 (12.2)	3.1 (3.7)	28 (5)	12	Gain incentive	$172
**[58]**Volpp 2008a	Obesity	Hospital	10	104.8 (14.3)	1.8 (4.1)	19	110.0 (13.7)	6.0 (5.7)	Not reported	16	Lottery	$447
**[58]**Volpp 2008b	Obesity	Hospital	9	104.8 (14.3)	1.8 (4.1)	19	109.5 (11.3)	6.4 (4.6)	Not reported	16	Deposit contract	$1,225
**[45]**Almeida 2015**	Obesity	Workplace	1001	95.4 (21.1)	0.6 (16.1)	789	93.7 (20.0)	1.0 (15.5)	48 (3)	24	Gain incentive	Not reported

*N reflects splitting of control group sample size across multiple arms from the same trial, where applicable

**Outlying study excluded from sensitivity analysis.

FI: financial incentives; OA: osteoarthritis; SD: standard deviation

#### Quality assessment

We present the methodological quality and reporting of the 11 included trials as assessed using Jadad score in the Supporting Information (Table C in S1 File).[41] The median Jadad score was 4, ranging from 3 to 5 out of 5.

#### Intervention characteristics

Of the 11 included studies (19 intervention arms), two studies (two intervention arms) intended to increase PA,[47, 50] while nine studies (17 intervention arms) aimed to promote WL.[45, 51–57] Of 19 FI intervention arms in 11 studies, eight had gain incentive schemes,[45, 47, 50, 51, 55, 56] two implemented loss incentive schemes,[53] six utilized deposit contracts,[52, 54, 58] two used lottery schemes,[56, 58] and one used a combination of gain incentive and lottery.[51] The derivation of the total amount of FI a subject could earn, adjusted to 2016 USD, is described in the Supporting Information (Table D in S1 File). The median of the possible FI amount per week or the expected value per week for lottery incentive arms, in 2016 inflation-adjusted USD, was $22.41 (25^th^– 75^th^ percentile: $12.36 - $36.37), ranging from $10.72 to $76.54. Almeida (2015) did not report the actuarial amount of FI offered to participants. The mean primary follow-up time point was at 24.7 (SD: 10.6) weeks, ranging from 12 to 52 weeks.

### Effect of financial incentives on physical activity and weight loss

**Fig 3:**
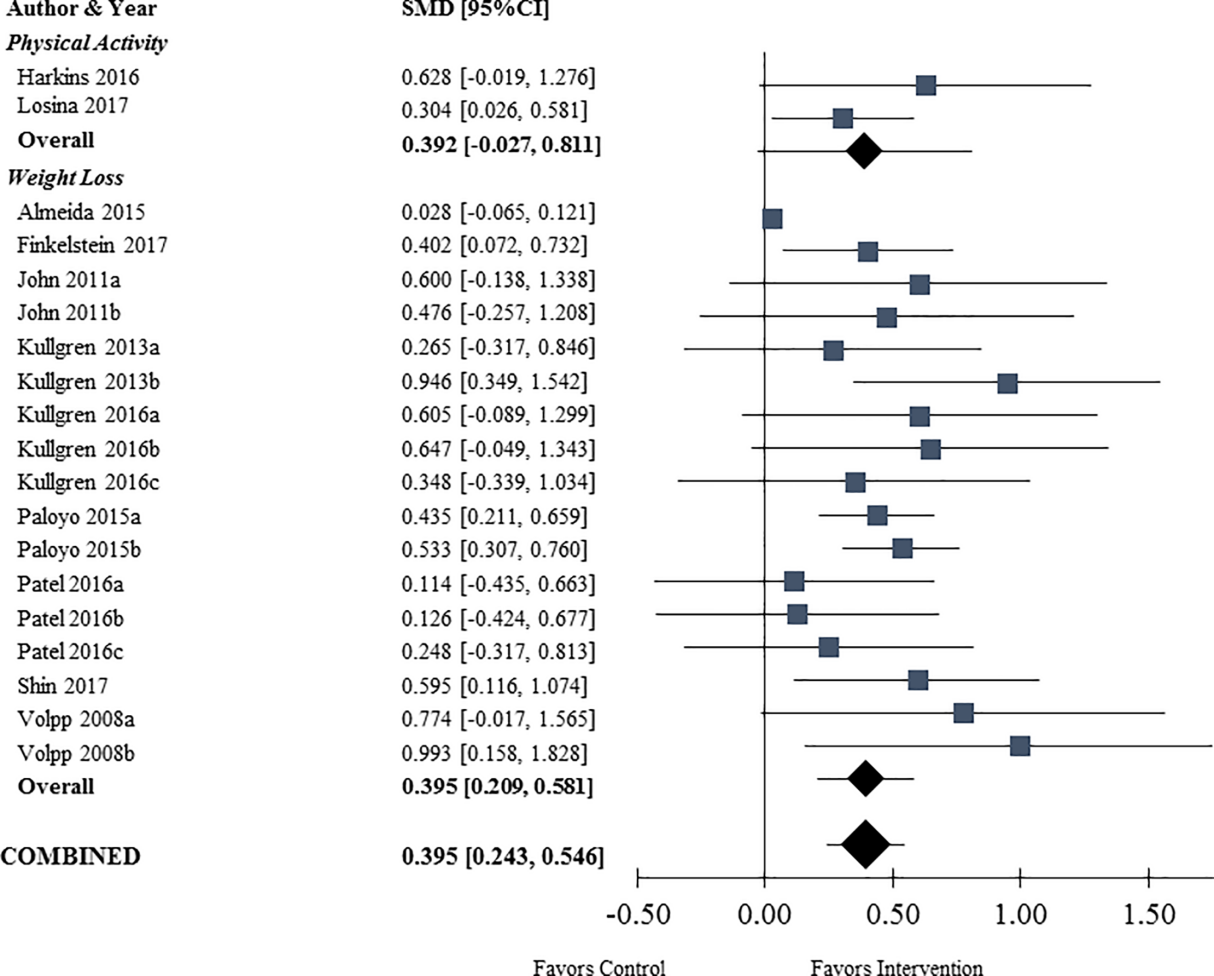
Forest plot of standardized mean differences (SMDs) (n = 11 studies, 19 study arms). Each study arm is listed on the y-axis. Study arms are grouped by outcome type (PA or WL). The SMD is along the x-axis with a vertical line at zero representing the null value. Squares represent study arm-specific SMDs. Diamonds represent combined estimates from the random effects meta-analysis. The small diamonds represent the combined estimates for each outcome type (PA or WL) separately. The large diamond represents the overall combined estimate of all study arms for both outcomes. Bars are 95% confidence intervals.

In the two studies measuring PA outcomes, the pooled mean difference in steps per day was 940 (95%CI [306–1574]) steps (S1 Fig), translating to a pooled SMD of 0.392 (95%CI [-0.027–0.811]). In the nine studies measuring WL outcomes, the pooled mean difference in kilograms lost was 2.32 (95%CI [1.76–2.88]), translating to a pooled SMD of 0. 395 (95%CI [0.209–0.581]) (S2 Fig). Both outcomes favored FI interventions over control (Fig 3). The pooled SMD of all outcomes for treatment compared to control was 0.395 (95%CI [0.243–0.546]; p<0.001), indicating a moderate, statistically significant effect on PA and WL in those who received performance-contingent FI compared to control participants.

In a sensitivity analysis excluding the study with high contribution to overall heterogeneity, the pooled mean difference for the eight studies measuring WL outcomes was 2.36 kg (95% CI [1.80–2.93]), translating to a pooled SMD of 0.562 (95% CI [0.296–0.827]). The pooled SMD of all outcomes for treatment compared to control was 0. 452 (95%CI [0. 351–0. 554]; p<0.001).

**Fig 4:**
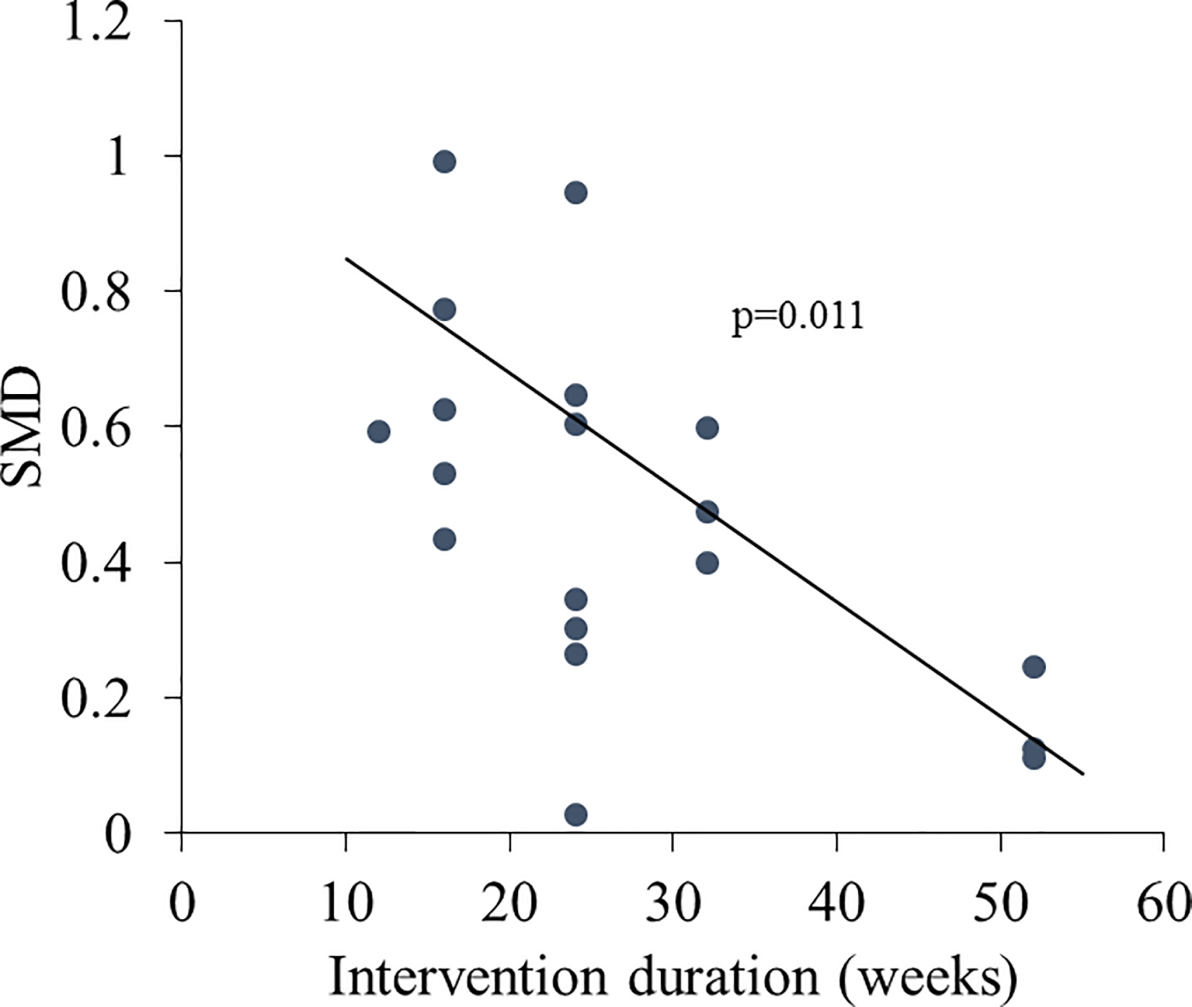
Correlation between length of intervention and SMD. SMD is along the y-axis. Intervention duration, in weeks, is the independent variable. Each dot represents a study arm. The lines are generated from random effects meta-regressions between intervention duration and SMD.

Longer interventions appeared to be associated with lower SMD: a 10-week increase in intervention duration was associated with a 0.177 decrease in SMD (p = 0.011; Fig 4). We did not find significant associations between the outcome and any other covariate.

Differences between control arm designs significantly contributed to the outcome. The frequency of interaction with a health counselor (p<0.001) and the frequency of following up with control arm participants (p = 0.005) affected the difference in outcomes between the control arm and the intervention arms. We used a multivariate meta-regression to determine the effect of both the control arm characteristics. The effects of both the frequency of counseling and the follow-up frequency of the control arm remained significant (p = 0.001 and 0.04, respectively); regular counseling and less than monthly follow-up in the control arm was associated with the lowest SMD. These results were similar in a model additionally adjusting for outcome type (PA vs. WL). In sensitivity analysis excluding Almeida (2015),[45] which had high weight and contributed significantly to heterogeneity, the frequency in which the control arm receiving coaching or were followed up by the study were not associated with effect (p = 0.533 and 0.402, respectively).

## Discussion

To our knowledge, this is the first meta analysis evaluating the impact of FI on objectively-measured PA and WL outcomes. Our results suggest that FI are moderately effective in improving PA and WL in adults with sedentary lifestyles or comorbidities, increasing steps per day by 940 steps and weight loss **by 2.3** kilograms compared to control participants. We found that greater weekly actuarial FI and shorter interventions were positively associated with favorable outcomes.

Several meta-analyses on FI for a range of health behavior changes have been conducted and have varied conclusions. One evaluated the effect of FI on PA found that incentives increased exercise attendance by 11.6% when compared to control.[32] In a meta-analysis on FI for behavioral obesity treatments, there were no significant effects of FI compared to control.[24] For PA, FI was effective if dispersed conditionally upon meeting goals, but not when given unconditionally, such as a gym membership.[26] Another meta-analysis found that FI for smoking cessation, vaccination or screening attendance, and physical activity was more effective than usual care or no intervention, with an effect size of 1.62 for all health-related outcomes.[23] Additionally, these studies included self-reported outcomes, which may bias results in trials[33, 59] because people tend to over-report PA levels.[59, 60] Ours was the first meta-analysis to look at objectively-measured PA and WL outcomes exclusive of other health-related measures.

The SMD for WL that we report in the current analysis is **0.395**, which is in line with the effect size of 0.47 reported in a meta-analysis of motivational interviewing (MI), another behavioral intervention, for WL.[61] For PA, we calculated an effect size of **0.392**, which is higher than that found in meta-analyses of other behavioral interventions; these meta-analyses reported effect sizes between 0.07 and 0.27 and included studies that included both self-reported and objective outcomes.[61–63] Extrinsic motivators, such as FI, may be more effective than intrinsic motivators when an individual has limited initial interest in pursuing an activity because extrinsic motivators offer rewards associated with an independent outcome;[64] this may explain the increased effectiveness of FI compared to MI in the short-term.

We found a smaller difference between the control arm and the intervention arm when the control group had more frequent interactions with a health coach. Control participants who receive coaching may be more motivated to participate in healthy behaviors, likely causing a smaller difference seen between control and intervention groups in those studies. Another meta-analysis found that in WL studies, control groups receiving no intervention lost less weight compared to groups that received usual care for WL, which generally includes health coaching.[65] We also found a smaller difference between the control arm and the intervention arm when the control group had less frequent follow-up about their objective outcomes; however, in the sensitivity analysis excluding Almeida (2015), the study that contributed significantly to heterogeneity due to high weight, both these control arm characteristics were not associated with intervention effect.

We found that longer intervention duration is associated with an attenuated intervention effect. This may be due to external rewards providing diminishing motivation over time as an individual’s focus shifts from executing activities to earning rewards.[64] When designing FI interventions, habit formation should be emphasized over rewards. Our analysis did not evaluate sustainability of interventions due to the heterogeneity of secondary follow-up time points across studies. Maintenance of the favored behavior is a crucial goal of any intervention, and this underscores the need to evaluate intervention sustainability in future FI studies.

### Limitations

The results of this analysis should be viewed considering several limitations. Trials of FI to improve PA or WL reported dissimilar outcomes, such as steps per day and WL in kilograms or pounds. In addition to considering PA and WL outcomes separately, we compared data across both outcomes. While we attempted to do this by using SMDs,[46] it is possible that the outcome measures may be associated with different underlying constructs. The SMD for overall effect allows for comparison across multiple measurement scales but does not capture potential differences in study populations, such as variation in participant characteristics for those engaging in PA versus WL studies.

While we assessed study quality with the Jadad score, we did not exclude studies based on quality. Further, a challenge in interpreting FI intervention outcomes is that the comparator arms often received heterogeneous treatments. We found significant associations between characteristics of the control arm and treatment effect, whereby less intensive control arms (less frequent coaching and follow-up) were associated with higher treatment effect. Further, while we did not find significant associations between many FI characteristics and outcome, there was heterogeneity within scheme types; for example, of the six intervention arms using deposit contracts, only one was unmatched[54] and the other five increased possible rewards with 1:1 or 2:1 matching.[52, 54, 58] These differences in implementation may influence intervention effects. While meta-analyses can be conducted on as few as two studies, our sample size had 11 studies with **19** intervention arms, limiting our ability to conduct subgroup analyses, thus our analyses were likely underpowered to detect differences in effect by study characteristics.[66] Finally, previous works have suggested that individual- or population-level demographic characteristics may affect the efficacy of FI interventions,[29, 55, 67] and this should be examined in future studies on FI.

## Conclusions

We found a statistically significant estimate that subjects in groups receiving FI averaged 940 (95% CI [306–1,574]) more steps after PA interventions and lost on average 2.32 (95% CI [1.76–2.88]) more kilograms after WL interventions. Overall, we found a combined SMD of 0.395 (95%CI [0.243–0.546]; p<0.001) favoring FI over control for improving PA and WL outcomes. This meta-analytic review supports the use of FI in interventions to improve PA or WL. This analysis underscores the need for further studies to determine the sustainability of FI interventions; which subgroups, if any, benefit the most from FI; and which FI designs are most effective. These results may be useful for informing targeted interventions and policy decisions. Lifestyle improvement and public health programs should consider incorporating FI or pairing FI with other behavioral interventions invoking intrinsic motivation to improve PA or WL outcomes.

## Supporting information

S1 Checklist PRISMA checklist. (DOC)

S1 File **Appendix with search strings run in PubMed, Embase, and Web of Science; Table A with list of authors consults and data included in analysis; Table B with detailed summaries of included papers; Table C with methodological quality assessment: Jadad scores.**[41]; Table D with derivation of maximum weekly possible financial incentives values.
(DOCX)

S1 Fig Forest plot of primary meta-analysis of steps per day. (TIF)

S2 Fig Forest plot of primary meta-analysis of weight loss. (TIF)
