# Supplementary material for: Financial incentives for objectively-measured physical activity or weight loss in adults with chronic health conditions: A meta-analysis
Source: PLoS One. 2018 Sep 25;13(9):e0203939. doi: 10.1371/journal.pone.0203939 (PMC6156024; doi:10.1371/journal.pone.0203939)
Supplement: S1 File — Appendix with search strings run in PubMed, Embase, and Web of Science; Table A with list of authors consults and data included in analysis; Table B with detailed summaries of included papers; Table C with methodological quality assessment: Jadad scores.[41]; Table D with derivation of maximum weekly possible financial incentives values. (DOCX) [file pone.0203939.s002.docx]

Financial incentives for objectively-measured physical activity or weight loss in adults with chronic health conditions: a meta-analytic systematic review

**Supporting Information (S1 File)**

**Appendix.** Search strings run in PubMed, Embase, and Web of Science.

**Table A.** List of authors consulted and data included in analysis.

**Table B.** Detailed summaries of included papers.

**Table C.** Methodological quality assessment: Jadad scores.[[1](#_ENREF_1)]

**Table D.** Derivation of maximum weekly possible financial incentives values.

Supporting information is presented the in order of appearance in the manuscript.

**Appendix. Search strings run in PubMed, Embase, and Web of Science.**

All searches were run on June 27, 2017 and re-run on July 27, 2017 to capture articles published between June 27, 2017 and July 27, 2017.

1. **PubMed**:
   (achievement[mh] OR (financial incentive*) OR (financial reward*) OR lotter* OR (monetary incentiv*) OR (monetary reward*) OR economics, behavioral[mh] OR reimbursement, incentive[mh] OR reinforcement OR (loss aversion)) AND (weight reduction programs[mh] OR weight loss[mh] OR exercise[mh] OR (weight management) OR (physical activity) or (physically active)) AND (musculoskeletal diseases[mh] OR cardiovascular diseases[mh] OR overweight[mh] OR diabetes mellitus[mh] OR chronic disease[mh] OR sedentary lifestyle[mh] OR inactiv*)

   Results (June 27, 2017): 620
   Results (January 01, 2017 to July 27, 2017): 188
2. **Embase**:
   TS=((goal attain* OR achievement OR “financial incentive*” OR “financial reward*” OR lotter* OR “monetary incentiv*” OR “monetary reward*” OR “behavio*ral economic*” OR incentive* OR reinforcement OR “loss aversion”) AND (“weight reduction” OR “weight loss” OR exercise OR “weight management” OR “physical* activ*”) AND (“musculoskeletal disease*” OR “cardiovascular disease*” OR overweight OR obes* OR diabetes OR “chronic disease*” OR sedentary or inactive*))

   Results (June 27, 2017): 714
   Results (June 29, 2017 to July 27, 2017): 7
   *Note*: In Embase, we re-ran the search using the timeframe “over previous four weeks.”
3. **Web of Science**:
   'goal attainment'/exp OR 'lottery' OR 'monetary reward'/exp OR 'incentives'/exp OR 'incentive'/exp OR 'financial incentive'/exp OR 'behavioral economics'/exp OR 'reinforcement'/exp OR 'awards and prizes'/exp OR 'endowment effect'/exp AND ('body weight management'/exp OR 'weight reduction'/exp OR 'weight management'/exp OR 'exercise'/exp OR 'physical activity'/exp) AND ('musculoskeletal disease'/exp OR 'cardiovascular disease'/exp OR 'chronic disease'/exp OR 'obesity'/exp OR 'diabetes mellitus'/exp OR 'sedentary lifestyle'/exp OR 'sedentary behavior'/exp OR 'physical inactivity'/exp)

   Results (June 27, 2017): 2,756
   Results (June 27, 2017 to July 27, 2017): 18

**Table A. List of authors consulted and data included in analysis.**

| **Author Names** | **Ref** | **Date of initial correspondence** | **Date data received** | **Data from authors included in analysis** |
| --- | --- | --- | --- | --- |
| Alfredo Paloyo, Ph.D. | [[2](#_ENREF_2)] | May 16, 2017 | June 18, 2017 | Means and standard deviations by study arm used to generate Figure 1 of the Paloyo et al. (2015) published manuscript. |
| Karen Glanz, M.P.H.  Kristin Harkins, B.A.  Jason Karlawish, M.D. | [[3](#_ENREF_3)] | July 12, 2017 | August 09, 2017 | Weekly means and standard deviations in change in daily steps per week at week 16; these data were used to generate Figure 1 in the Harkins et al. (2017) published manuscript. |

**Table B.** **Detailed summaries of included papers.**

| **Study** | **Participants** | **Intervention Group(s)** | **Control Group** |
| --- | --- | --- | --- |
| [[4](#_ENREF_4)]Almeida et al. (2015) | **Health condition:** Obesity  **Source of recruitment:** Workplace  **Outcome measure:** Weight loss  - Goal: Loss of a modest percentage of body weight each month  **Primary outcome time point:** 24 weeks | ***(Intervention 1: Gain Incentive)***  **N:** 789  **Mean age (SD):** 45.68 (3.30)  **Intervention:**  - Participants received $1 each month for every percentage of body weight lost  - Website option for participants to log PA and dietary intake  ***Maximum total expected value (2016USD):** Not reported  **Maximum weekly expected value (2016USD):** Not reported  ($1 per percentage of body weight lost). | ***(Control Arm)***  **N:** 1001  **Mean age (SD):** 48.24 (2.78)  **Control Arm:**  - 4 newsletters, delivered quarterly, provided information on exercise, eating plans, and meal ideas.  - Four one-hour in-person resource sessions were offered. These included information on health and nutrition. |
| [[5](#_ENREF_5)]Finkelstein et al. (2017) | **Health condition:** Obesity  **Source of recruitment:** Community  **Outcome measure:** Weight loss (primary) and physical activity (secondary)  - Goal: lose 8% baseline weight in 8 months  **Primary outcome time point:** 32 weeks | ***(Intervention 1 - Gain Incentive and Lottery Incentive)***  **N:** 107  **Mean age (SD):** 43.4 (9.8)  **Gain Incentive:**  - Obesity management (OBM) program plus S$20 monthly contingent on losing 1 kg from previous payout or BMI <23.5kg/m^2^  - Received an additional S$20 per month for logging ≥10,000 steps/day on pedometer on ≥20 days per month  - Bonus S$100 contingent on losing at least 5% of baseline weight or BMI <23.5 kg/m^2^ at 4-month weigh-in  - Bonus S$200 contingent on losing at least 8% of baseline weight or BMI <23.5 kg/m^2^ at 8-month weigh-in  **Lottery Incentive**  - OBM plus same reward scheme as above with a 10% chance of winning 10 times the amount and a 90% chance of no reward  ***Maximum total expected value (2016USD):** $384.60  **Maximum weekly expected value (2016USD):** $12.02 | ***(Control Arm)***  **N:** 54  **Mean age (SD):** 45.0 (10.2)  **Control Arm:**  - 4-month program that teaches subjects skills required to maintain healthy lifestyle  - Program includes medical management, behavioral modification, diet and nutrition management, and individualized exercise prescription |
|  |  |  |  |
| [[3](#_ENREF_3)]Harkins et al. (2017) | **Health condition:** Sedentary lifestyle  **Source of recruitment:** Community  **Outcome measure:** Physical activity  - Goal: 50% increase in daily steps on ≥5 days per week  **Primary outcome time point:** 16 weeks | ***(Intervention - Gain Incentive)***  **N:** 24  **Mean age (SD):** 79.5 (5.8)  **Intervention:**  - Automated e-mail feedback about progress received every week  - Received $20 for each week that their walking goal was met for ≥5 of 7 days  ***Maximum total expected value (2016USD):** $329.68  **Maximum weekly expected value (2016USD):** $20.61 | ***(Control Arm)***  **N:** 16  **Mean age (SD):** 79.7 (6.8)  **Control Arm:**  - Wore pedometers every day and uploaded data every night  - Automated e-mail feedback about progress sent to subjects weekly |
| **Study** | **Participants** | **Intervention Group(s)** | **Control Group** |
| [[6](#_ENREF_6)]John et al. (2011) | **Health condition:** Obesity  **Source of recruitment:** Hospital  **Outcome measure:** Weight loss  - Goal: lose 24 lb in 24 weeks, maintain weight lost for 8 additional weeks  **Primary outcome time point:** 32 weeks | ***(Intervention 1 - Deposit Contract 1)***  **N:** 22  **Mean age (SD):** Not reported  **Intervention:**  - Opportunity to deposit $0.00-$3.00 per day, which would be refunded and matched 1:1 every day that the subject self-reports meeting weight loss (WL) goal. Receiving the reward is contingent upon meeting WL goal at the end of each month as measured during a weigh-in  - First 24 weeks framed as "weight loss period;" final 8 weeks framed as "maintenance of weight loss period"  ***Maximum total expected value (2016USD):** $1,645.13  **Maximum weekly expected value (2016USD):** $51.41 | ***(Control Arm)***  **N:** 22  **Mean age (SD):** Not reported  **Control Arm:**  - Initial 1-hour one-on-one consultation with dietitian to discuss weight loss strategies |
|  |  | ***(Intervention 2 - Deposit Contract 2)***  **N:** 22  **Mean age (SD):** Not reported  **Intervention:**  - Deposit contract scheme same as above; however, no distinction between first 24 weeks and final 8 weeks  ***Maximum total expected value (2016USD):** $1,645.13  **Maximum weekly expected value (2016USD):** $51.41 |  |
| [[7](#_ENREF_7)]Kullgren et al. (2013) | **Health condition:** Obesity  **Source of recruitment:** Workplace  **Outcome measure:** Weight loss  - Goal: lose 0.45 kg per week for 24 weeks  **Primary outcome time point:** 24 weeks | ***(Intervention 1 - Individual, Loss Incentives)***  **N:** 35  **Mean age (SD):** 44.4 (11)  **Intervention:**  - Subjects told of $100 set aside for them each month that they would receive if they met their weight loss goals at the weigh-in  ***Maximum total expected value (2016USD):** $640.19  **Maximum weekly expected value (2016USD):** $26.67 | ***(Control Arm)***  **N:** 35  **Mean age (SD):** 44.5 (10)  **Control Arm:**  - Provided a link to information on weight-control |
|  |  | ***(Intervention 2 - Group, Loss Incentives)***  **N:** 35  **Mean age (SD):** 47.0 (9)  **Intervention:**  - Participants placed into groups of 5 with identities of group members unknown  - Told that at the end of each month, $500 would be split among group members that met their weight loss goals at weigh-in  ***Maximum total expected value (2016USD):** $640.19  **Maximum weekly expected value (2016USD):** $26.67 |  |
| **Study** | **Participants** | **Intervention Group(s)** | **Control Group** |
| [[8](#_ENREF_8)]Kullgren et al. (2016) | **Health condition:** Obesity  **Source of recruitment:** Workplace  **Outcome measure:** Weight loss  - Goal: lose 1 lb per week for 24 weeks  **Primary outcome time point:** 24 weeks | ***(Intervention 1 - Deposit Contract, No Match)***  **N:** 33  **Mean age (SD):** 43.5 (9.6)  **Intervention:**  - Provided link to health website and attended monthly weigh-ins  - Opportunity to deposit $1-$3 per day. Each day that participants weighed at or below goal, the money was returned to the participant  ***Maximum total expected value (2016USD):** $537.76  **Maximum weekly expected value (2016USD):** $22.41  ***(Intervention 2 - Deposit Contract, 1:1 Match)***  **N:** 33  **Mean age (SD):** 44.3 (11.5)  **Intervention:**  - Same as Intervention 1, but money returned was matched by the program 1:1  ***Maximum total expected value (2016USD):** $1,075.52  **Maximum weekly expected value (2016USD):** $44.81 | ***(Control Arm)***  **N:** 33  **Mean age (SD):** 45.9 (9.0)  **Control Arm:**  - Provided link to health website and attended monthly weigh-ins |
|  |  | ***(Intervention 3 - Deposit Contract, 2:1 Match)***  **N:** 33  **Mean age (SD):** 42.0 (9.6)  **Intervention:**  - Same as Intervention 1, but money returned was matched by the program 2:1  ***Maximum total expected value (2016USD):** $1,613.28  **Maximum weekly expected value (2016USD):** $67.22 |  |
|  |  |  |  |
| **Study** | **Participants** | **Intervention Group(s)** | **Control Group** |
| [[9](#_ENREF_9)]Losina et al. (2017) | **Health condition:** Post-total knee replacement for osteoarthritis  **Source of recruitment:** Hospital  **Outcome measure:** Physical activity  - Goal: 50% increase in step count from baseline or ≥150 minutes of MVPA  **Primary outcome time point:** 24 weeks | ***(Intervention 1 - Gain Incentive)***  **N:** 102  **Mean age (SD):** 65 (8)  **Intervention:**  - Subjects were asked to complete daily physical activity logs from weeks 2-8 and weekly logs from weeks 9-23  - Subjects received $5 for completing ≥5/7 daily logs or a weekly log  - $15 bonus payment for each weekly that self-reported minutes of physical activity (Yale Physical Activity Scale) increased by ≥10% from previous week  - Subjects wore accelerometer at 3 and 6 months for one week  - $50 bonus for increasing daily steps by at least 50% from 3 to 6 months or met PA guidelines of ≥150 minutes of MVPA per week  ***Maximum total expected value (2016USD):** $305.00  **Maximum weekly expected value (2016USD):** $12.71 | ***(Control Arm)***  **N:** 100  **Mean age (SD):** 65 (7)  **Control Arm:**  - Received attention control calls about general health messages and to answer questions about the study |
| [[2](#_ENREF_2)]Paloyo et al. (2015) | **Health condition:** Obesity  **Source of recruitment:** Rehabilitation clinic  **Outcome measure:** Weight loss  - Goal: lose 6-8% of starting weight in four months  **Primary outcome time point:** 16 weeks | ***(Intervention 1 - Gain Incentive €150)***  **N:** 237  **Mean age (SD):** 48 (9)  **Intervention:**  - €150 received if subjects met weight loss target at the end of the intervention  - If subjects met <50% of weight loss goal, they received no reward. If the ≥50% threshold was reached, the €150 was disbursed proportionally  ***Maximum total expected value (2016USD):** $213.97  **Maximum weekly expected value (2016USD):** $13.37 | ***(Control Arm)***  **N:** 234  **Mean age (SD):** 48(10)  **Control Arm:**  - Received medical advice and counseling |
|  |  | ***(Intervention 2 - Gain Incentive €300)***  **N:** 229  **Mean age (SD):** 47 (9)  **Intervention:**  - €300 received if subjects met weight loss target at the end of the intervention  - If subjects met <50% of weight loss goal, they received no reward. If the ≥50% threshold was reached, the €300 was disbursed proportionally  ***Maximum total expected value (2016USD):** $427.94  **Maximum weekly expected value (2016USD):** $26.75 |  |
| **Study** | **Participants** | **Intervention Group(s)** | **Control Group** |
| [[10](#_ENREF_10)]Patel et al. (2016) | **Health condition:** Obesity  **Source of recruitment:** Workplace  **Outcome measure:** Weight loss  - Goal: Lose 5% of starting weight over 6 months and maintain weight loss over subsequent 6 months  **Primary outcome time point:** 52 weeks | ***(Intervention 1 - Delayed Adjustment)***  **N:** 51  **Mean age (SD):** 45.1 (9.9)  **Intervention:**  - If subject met 5% weight loss target, they would receive $550 in 26 bi-weekly premium discounts starting in the year after the intervention ends  ***Maximum total expected value (2016USD):** $557.60  **Maximum weekly expected value (2016USD):** $10.72 | ***(Control Arm)***  **N:** 50  **Mean age (SD):** 44.9 (10.6)  **Control Arm:**  - Attended baseline, 6- and 12-month weigh-ins |
|  |  | ***(Intervention 2 - Immediate Adjustment)***  **N:** 50  **Mean age (SD):** 45.7 (9.5)  **Intervention:**  - If subject met 5% weight loss target, they would receive $550 in 26 bi-weekly premium discounts immediately  ***Maximum total expected value (2016USD):** $557.60  **Maximum weekly expected value (2016USD):** $10.72 |  |
|  |  | ***(Intervention 3 - Daily Lottery)***  **N:** 50  **Mean age (SD):** 43.9 (9.2)  **Intervention:**  - Given daily weight target (overall goal of 5% loss in first 6 months and maintenance in last 6 months)  - Subjects had 18% chance of winning $10 and 1% chance of winning $100; expected value = $2.80 daily.  - Money can only be collected if subject achieved target weight confirmed during weigh-in  ***Maximum total expected value (2016USD):** $577.60  **Maximum weekly expected value (2016USD):** $10.72 |  |
|  |  |  |  |
|  |  |  |  |
|  |  |  |  |
| **Study** | **Participants** | **Intervention Group(s)** | **Control Group** |
| [[11](#_ENREF_11)]Shin et al. (2017) | **Health condition:** Obesity  **Source of recruitment:** University  **Outcome measure:** Weight loss  - Goal: lose 7% of baseline weight at 12 weeks  **Primary outcome time point:** 12 weeks | ***(Intervention 1 – Gain Incentive)***  **N:** 35  **Mean age (SD):** 28.1 (6.0)  **Intervention:**  - Smartcare intervention plus financial incentives contingent on daily physical activity and weight loss goals  - Physical activity goals: 1,000 KRW per day of meeting goals; additional 3,000 KRW per full week of meeting goals  - Weight loss goals: 50,000 KRW for achievement of 3% and 5% weight loss from baseline at 4 and 8 weeks; 100,000 KRW for achieving 7% at week 12  - Physical activity rewards paid out at end of the study; weight loss rewards paid out after meeting goals at each visit  ***Maximum total expected value (2016USD):** $171.76  **Maximum weekly expected value (2016USD):** $14.31 | ***(Control Arm)***  **N:** 35  **Mean age (SD):** 28.7 (4.6)  **Control Arm:**  - Smartcare intervention group: received accelerometer and smartphone application that synchronizes accelerometer data via Bluetooth |
| [[12](#_ENREF_12)]Volpp et al. (2008) | **Health condition:** Obesity  **Source of recruitment:** Hospital  **Outcome measure:** Weight loss  - Goal: lose 1 lb per week for 16 weeks  **Primary outcome time point:** 16 weeks | ***(Intervention 1: Lottery Incentive)***  **N:** 19  **Mean age (SD):** Not reported  **Intervention:**  - Eligible for daily lottery contingent upon meeting weight loss goal  - Lottery scheme: 1 in 100 chance of $100 reward, 1 in 5 chance of $10 reward  ***Maximum total expected value (2016USD):** $446.81  **Maximum weekly expected value (2016USD):** $27.93 | ***(Control Arm)***  **N:** 19  **Mean age (SD):** Not reported  **Control Arm:**  - Initial 1-hour one-on-one consultation with dietitian to discuss weight loss strategies |
|  |  | ***(Intervention 2: Deposit Contract)***  **N:** 19  **Mean age (SD):** Not reported  **Intervention:**  - Opportunity to deposit $0.01-$3.00 per day, which would be refunded and matched 1:1 every day that the subject self-reports meeting weight loss goal. Receiving the reward was contingent upon meeting weight loss goal at the end of each month measured during a weigh-in  ***Maximum total expected value (2016USD):** $1,224.68  **Maximum weekly expected value (2016USD):** $76.54 |  |
|  | | | |

**Table C. Methodological quality assessment scores: Jadad scores.[**[**1**](#_ENREF_1)**]**

| **Study** | **Study described as randomized (0/1)** | **Randomization described and appropriate (0/1)** | **Study mentions blinding**  **(0/1)** | **Blinding method appropriate**  **(0/1)** | **Withdrawals described (0/1)** | **Randomization method inappropriate**  **(0/-1)** | **Blinding method inappropriate (0/-1)** | **Jadad Score** |
| --- | --- | --- | --- | --- | --- | --- | --- | --- |
| Almeida et al., 2015[[4](#_ENREF_4)] | 1 | 1 | 0 | 0 | 1 | 0 | 0 | 3 |
| Finkelstein et al., 2017[[5](#_ENREF_5)] | 1 | 1 | 1 | 1 | 1 | 0 | 0 | 5 |
| Harkins et al., 2017[[3](#_ENREF_3)] | 1 | 1 | 1 | 1 | 1 | 0 | 0 | 5 |
| John et al., 2011[[6](#_ENREF_6)] | 1 | 1 | 1 | 0 | 1 | 0 | -1 | 3 |
| Kullgren et al., 2013[[7](#_ENREF_7)] | 1 | 1 | 1 | 1 | 1 | 0 | 0 | 5 |
| Kullgren et al., 2016[[8](#_ENREF_8)] | 1 | 1 | 1 | 1 | 1 | 0 | 0 | 5 |
| Losina et al., 2017[[9](#_ENREF_9)] | 1 | 1 | 0 | 0 | 1 | 0 | 0 | 3 |
| Paloyo et al., 2015[[2](#_ENREF_2)] | 1 | 1 | 1 | 1 | 1 | 0 | 0 | 5 |
| Patel et al., 2016[[10](#_ENREF_10)] | 1 | 0 | 1 | 1 | 1 | 0 | 0 | 4 |
| Shin et al., 2017[[11](#_ENREF_11)] | 1 | 1 | 0 | 0 | 1 | 0 | 0 | 3 |
| Volpp et al., 2008[[12](#_ENREF_12)] | 1 | 1 | 1 | 0 | 1 | 0 | -1 | 3 |

**Table D. Derivation of maximum weekly possible financial incentives values.**

| **Author** | **Year** | **Arm** | **Description** | **FI Value (reported)** | **Currency conversion** | **Final year enrollment** | **2016 USD** |
| --- | --- | --- | --- | --- | --- | --- | --- |
| Volpp | 2008 | Deposit contract | *Deposit contract financial incentive participants were given the opportunity to contribute between $0.01 and $3.00 for each day of the month that were refundable at the end of the month if they met or exceeded their weight loss goal. 16 week (4 mo.) intervention.* | $ 336.00 |  |  |  |
|  |  |  | *As an incentive for participants to contribute to deposit contracts, we […] added a fixed payment of $3 per day.* | $ 336.00 |  |  |  |
|  |  |  | *We matched their money 1:1.* | $ 336.00 |  |  |  |
|  |  |  | *[All] participants in the lottery and deposit contracts who lost more than 20 lb by the end of the 4 months received a bonus of $50.* | $ 50.00 |  |  |  |
|  |  |  | Personal contributions with matching | **$ 1,058.00** |  | 2007 | **$1,224.68** |
| Volpp | 2008 | Lottery | *Participants in the lottery incentive group were eligible for a daily lottery prize with an expected value of $3/d. 16 week (112 d) intervention.* | $ 336.00 |  |  |  |
|  |  |  | *All participants in the lottery and deposit contracts who lost more than 20 lb by the end of the 4 months received a bonus of $50.* | $ 50.00 |  |  |  |
|  |  |  |  | **$ 386.00** |  | 2007 | **$ 446.81** |
| John | 2011 | DC1 | *DC participants could contribute between $0.00-$3.00 per day of their own funds to a deposit contract. 24 week WL intervention.* | $ 504.00 |  |  |  |
|  |  |  | *Participants in the DC groups who had lost at least 20 pounds by the end of the 32 weeks each received $467.80 (their share of the money forfeited by participants in the DC arms who did not attain their goals).* | $ 467.80 |  |  |  |
|  |  |  | Personal contribution without matching | $ 971.80 |  |  |  |
|  |  |  | Personal contributions with matching | **$ 1,475.80** |  | 2008 | **$1,645.13** |
| John | 2011 | DC2 | *DC participants could contribute between $0.00-$3.00 per day of their own funds to a deposit contract. 24 week WL intervention.* | $ 504.00 |  |  |  |
|  |  |  | *Participants in the DC groups who had lost at least 20 pounds by the end of the 32 weeks each received $467.80 (their share of the money forfeited by participants in the DC arms…)* | $ 467.80 |  |  |  |
|  |  |  | Personal contribution without matching | $ 971.80 |  |  |  |
|  |  |  | Personal contributions with matching | **$ 1,475.80** |  | 2008 | **$1,645.13** |
| **Author** | **Year** | **Arm** | **Description** | **FI Value (reported)** | **Currency conversion** | **Final year enrollment** | **2016 USD** |
| Kullgren | 2013 | Individual incentive | *Individual-incentive participants…were also told that $100 would be set aside for them at baseline, 4 weeks, 8 weeks, 12 weeks, 16 weeks, and 20 weeks, and that the $100 would be electronically transmitted to them if they met or exceeded their target monthly weight loss as determined by their monthly weigh-in.* | **$ 600.00** |  | 2011 | **$ 640.19** |
| Kullgren | 2013 | Group incentive | *Similar to that in the individual-incentive group, the up-front allocation of incentives for meeting weight-loss goals was $100 per participant per month. At the end of each 4-week period during the 24-week intervention, $500 was split among participants in each group [of 5] who were at or below their monthly target weight.* | **$ 600.00** |  | 2011 | **$ 640.19** |
| Almeida | 2015 | Incentives | *The incentives were based on the percent of weight loss (e.g., 1% weight loss = $1.00)… through participants' quarterly weigh-ins.* | Not reported. (%WL max not reported) |  | 2008 | **Not reported.** |
| Paloyo | 2015 | EUR 150 | *Two treatment groups obtained a cash reward (EUR150 and EUR300…) for achieving an individually-assigned target weight within four months.* | € 150.00 | **$ 200.53** | 2011 | **$ 213.97** |
| Paloyo | 2015 | EUR 300 | *Two treatment groups obtained a cash reward (EUR150 and EUR300 within 237 and 229 participants, respectively) for achieving an individually-assigned target weight within four months.* | € 300.00 | **$ 401.07** | 2011 | **$ 427.94** |
| Kullgren | 2016 | Not matched | *Over 24 weeks, participants were asked to lose 24 pounds and randomized to monthly weigh-ins or daily weigh-ins with monthly opportunities to deposit $1 to $3 per day that was not matched, matched 1:1, or matched 2:1.* |  |  |  |  |
|  |  |  | Personal contribution (no matching) | **$ 504.00** |  | 2011 | **$ 537.76** |
| Kullgren | 2016 | Matched 1:1 | *"Over 24 weeks, participants were asked to lose 24 pounds and randomized to monthly weigh-ins or daily weigh-ins with monthly opportunities to deposit $1 to $3 per day that was not matched, matched 1:1, or matched 2:1."* |  |  |  |  |
|  |  |  | Personal contribution | $ 504.00 |  |  |  |
|  |  |  | Personal contribution with matching | **$ 1,008.00** |  | 2011 | **$1,075.52** |
|  |  |  |  |  |  |  |  |
| **Author** | **Year** | **Arm** | **Description** | **FI Value (reported)** | **Currency conversion** | **Final year enrollment** | **2016 USD** |
| Kullgren | 2016 | Matched 2:1 | *Over 24 weeks, participants were asked to lose 24 pounds and randomized to monthly weigh-ins or daily weigh-ins with monthly opportunities to deposit $1 to $3 per day that was not matched, matched 1:1, or matched 2:1.* |  |  |  |  |
| (*cont.*) |  |  | Personal contribution | $ 504.00 |  |  |  |
|  |  |  | Personal contribution with matching | **$ 1,512.00** |  | 2011 | **$1,613.28** |
| Patel | 2016 | Delayed premium | *Participants in the delayed premium adjustment group were informed that if they met the 5 percent weight loss target, they would receive $550 in the form of twenty-six biweekly premium discounts beginning the following year.* | **$ 550.00** |  | 2014 | **$ 557.60** |
| Patel | 2016 | Immediate premium | *Participants in the immediate premium adjustment group were informed that once they met their weight target, they would immediately begin receiving $550 in the form of twenty-six biweekly premium discounts.* | **$ 550.00** |  | 2014 | **$ 557.60** |
| Patel | 2016 | Lottery | *Participants in the daily lottery incentive group were given a daily weight target... Based on the expected value of the lottery (about $2.80 per daily weigh-in), the average time these employees were at work (about 3.75 days per week), and the study period (fifty-two weeks), the maximum value of this incentive was approximately equivalent to the $550 incentive offered in the other two intervention arms.* | **$ 550.00** |  | 2014 | **$ 557.60** |
| Finkelstein | 2017 | Incentives | *For each of four fortnightly weigh-ins from the start of the study through month 2, and for six monthly weigh-ins from months 3 through 8, participants earned S$20.00 each time their weight was at least 1 kg less than the last time they received a payout.* | $S 200.00 | $ 151.75 |  |  |
|  |  |  | *Participants earned an additional S$20.00 each month if they logged >=10,000 steps/day on the pedometer on at least 20 days of the calendar month.* | *$S 160.00* | *$ 121.40* |  |  |
|  |  |  | *Participants who achieved 5% or greater weight loss or a BMI value below 23.5 at the 4-month weigh-in received a bonus payment of S$100.* | $S 100.00 | $ 75.87 |  |  |
|  |  |  | *Participants who achieved 8% or greater weight loss or a BMI value below 23.5 at the 8-month weigh-in received a final bonus payment of S$20.00* | $S 200.00 | $ 151.75 |  |  |
|  |  |  | All incentives | $S 660.00 | $ 500.76 |  |  |
|  |  |  | Only weight-based incentives | $S 500.00 | **$ 379.36** | 2014 | **$ 384.60** |
|  |  |  |  |  |  |  |  |
| **Author** | **Year** | **Arm** | **Description** | **FI Value (reported)** | **Currency conversion** | **Final year enrollment** | **2016 USD** |
| Finkelstein | 2017 | Lottery | *Participants who chose the lottery were offered the same rewards scheme but each reward was a lottery ticket… Although the combined (expected) value of the rewards was $660 [same as the other incentive arm]…* |  |  |  |  |
| (*cont.*) |  |  | *All incentives* | *$S 660.00* | *$ 500.76* |  |  |
|  |  |  | Only weight-based incentives | $ 500.00 | **$ 379.36** | 2014 | **$ 384.60** |
| Harkins | 2017 | Incentives | *Financial incentive arm participants received…$20 each week that they met their walking goal on >=5 of the past 7 days. 16 week intervention.* | **$ 320.00** |  | 2013 | **$ 329.68** |
| Losina | 2017 | All FI arms | *In total, subjects [in a financial incentives arm: FI or FI+THC] were eligible to earn a maximum of $305 from the FI component over the study duration.* | **$ 305.00** |  | 2016 | **$ 305.00** |
| Shin | 2017 | Reward | *The total possible amount that could be earned for process incentives (process incentive: 1,000 KRW per day and an additional 3,000 KRW for a full week [7 days] of meeting goals) was 120,000 KRW…* | *₩ 120,000.00* | $ 101.77 |  |  |
|  |  |  | *…and 200,000 KRW for outcome incentives (outcome incentive: 50,000 KRW for achievement of the weight loss targets of 3% and 5% of baseline body weight at weeks 4 and 8, respectively, and 100,000 KRW for the achievement of the final target of 7% at week 12)...* | ₩ 200,000.00 | $ 169.62 |  |  |
|  |  |  | All incentives | ₩ 320,000.00 | $ 271.39 |  |  |
|  |  |  | Only weight-based incentives | ₩ 200,000.00 | **$ 169.62** | 2015 | **$ 171.76** |

| **1. Formatting Legend** | |  | |  |
| --- | --- | --- | --- | --- |
|  | *Italics* | Description of financial incentives taken from published manuscripts. | | |
|  | Grey | Financial incentive contingent upon meeting an outcome goal other than the outcome that we are reporting. | | |
|  | **Bold** | The values in our analysis prior to standardizing to 2016 USD. | | |
|  | **Bold & Underline** | The values in our analysis after standardizing to 2016 USD. | | |
|  |  |  | |  |
| **2. Conversion Rates - FC to USD ($)** | | | |  |
|  | **Currency** | **Year of distribution** | | **FC conversion to USD** |
|  | EUR (€) | 2011 | | 0.748 |
|  | KRW (₩) | 2015 | | 1179.128 |
|  | SGD (S$) | 2014 | | 1.318 |
|  | Source: | <https://www.irs.gov/individuals/international-taxpayers/yearly-average-currency-exchange-rates> [[13](#_ENREF_13)] | | |
|  |  |  | |  |
| **3. Inflation Rates (USD)** | | | Source: <https://www.bls.gov/cpi/#data>[[14](#_ENREF_14)] | |

**Formatting and conversion notes for Table D:**

| **Year** | **Rate** |  | **Year** | **Rate** |  | **Year** | **Rate** |  | **Year** | **Rate** |  | **Year** | **Rate** |
| --- | --- | --- | --- | --- | --- | --- | --- | --- | --- | --- | --- | --- | --- |
| 2007 | 207.342 |  | 2009 | 214.537 |  | 2011 | 224.939 |  | 2013 | 232.957 |  | 2015 | 237.017 |
| 2008 | 215.303 |  | 2010 | 218.056 |  | 2012 | 229.594 |  | 2014 | 236.736 |  | 2016 | 240.007 |

**SUPPLEMENTARY MATERIAL REFERENCES**

1. Jadad AR, Moore RA, Carroll D, Jenkinson C, Reynolds DJ, Gavaghan DJ, et al. Assessing the quality of reports of randomized clinical trials: is blinding necessary? Control Clin Trials. 1996;17(1):1-12.

2. Paloyo AR, Reichert AR, Reuss-Borst M, Tauchmann H. Who responds to financial incentives for weight loss? Evidence from a randomized controlled trial. Soc Sci Med. 2015;145:44-52.

3. Harkins KA, Kullgren JT, Bellamy SL, Karlawish J, Glanz K. A trial of financial and social incentives to increase older adults' walking. Am J Prev Med. 2017;52(5):E123-E30.

4. Almeida FA, You W, Harden SM, Blackman KC, Davy BM, Glasgow RE, et al. Effectiveness of a worksite-based weight loss randomized controlled trial: the worksite study. Obesity (Silver Spring). 2015;23(4):737-45.

5. Finkelstein EA, Tham KW, Haaland BA, Sahasranaman A. Applying economic incentives to increase effectiveness of an outpatient weight loss program (TRIO) – a randomized controlled trial. Soc Sci Med. 2017;185:63-70.

6. John LK, Loewenstein G, Troxel AB, Norton L, Fassbender JE, Volpp KG. Financial incentives for extended weight loss: a randomized, controlled trial. J Gen Intern Med. 2011;26(6):621-6.

7. Kullgren JT, Troxel AB, Loewenstein G, Asch DA, Norton LA, Wesby L, et al. Individual- versus group-based financial incentives for weight loss: a randomized, controlled trial. Ann Intern Med. 2013;158(7):505-14.

8. Kullgren JT, Troxel AB, Loewenstein G, Norton LA, Gatto D, Tao Y, et al. A randomized controlled trial of employer matching of employees' monetary contributions to deposit contracts to promote weight loss. Am J Health Promot. 2016;30(6):441-52.

9. Losina E, Collins JE, Deshpande BR, Smith SR, Michl GL, Usiskin IM, et al. Financial incentives and health coaching to improve physical activity following total knee replacement: a randomized controlled trial. Arthritis Care Res (Hoboken). 2017.

10. Patel MS, Asch DA, Troxel AB, Fletcher M, Osman-Koss R, Brady J, et al. Premium-based financial incentives did not promote workplace weight loss in a 2013-15 study. Health Aff (Millwood). 2016;35(1):71-9.

11. Shin DW, Yun JM, Shin JH, Kwon H, Min HY, Joh HK, et al. Enhancing physical activity and reducing obesity through smartcare and financial incentives: a pilot randomized trial. Obesity (Silver Spring). 2017;25(2):302-10.

12. Volpp KG, John LK, Troxel AB, Norton L, Fassbender J, Loewenstein G. Financial incentive-based approaches for weight loss: a randomized trial. JAMA. 2008;300(22):2631-7.

13. Internal Revenue Service. Yearly Average Currency Exchange Rates. [Web] Washington, D.C.2017 [updated August 17, 2017; cited 2018 January 8]; Available from: [www.irs.gov/individuals/international-taxpayers/yearly-average-currency-exchange-rates](file:///C:\Users\gs650\Downloads\www.irs.gov\individuals\international-taxpayers\yearly-average-currency-exchange-rates).

14. United States Department of Labor - U.S. Bureau of Labor Statistics. Consumer Price Index. 2017 [August 18, 2017]; Available from: [www.bls.gov/cpi/#data](file:///C:\Users\gs650\Downloads\www.bls.gov\cpi\#data).
