# Supplementary figures and images for: Financial incentives for objectively-measured physical activity or weight loss in adults with chronic health conditions: A meta-analysis
Source: PLoS One. 2018 Sep 25;13(9):e0203939. doi: 10.1371/journal.pone.0203939 (PMC6156024; doi:10.1371/journal.pone.0203939)

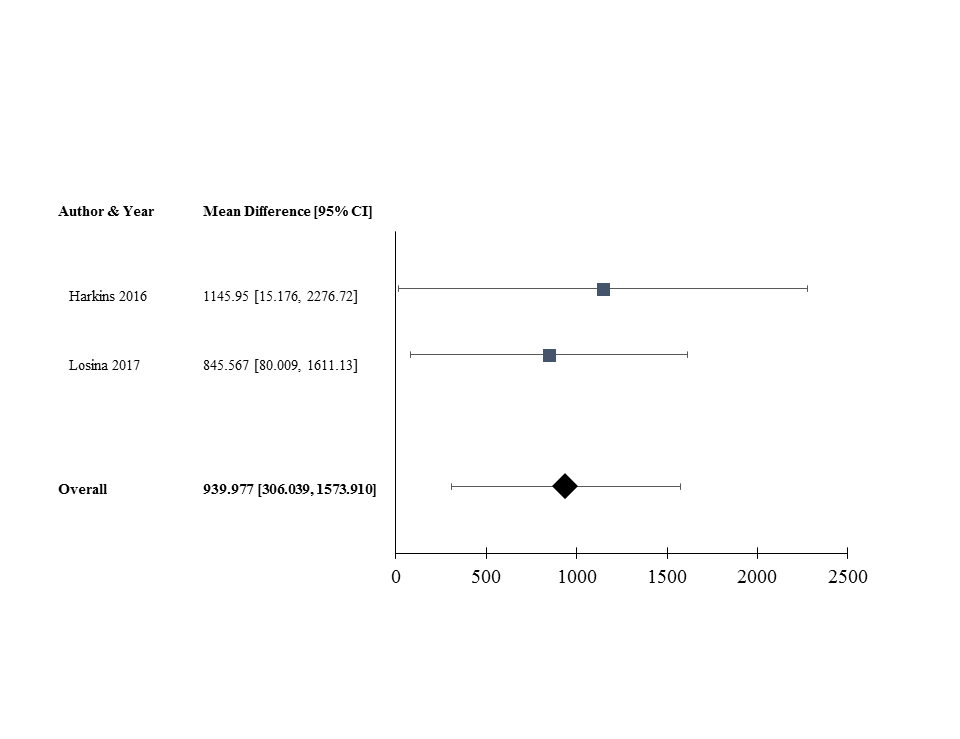

Supplement: S1 Fig — (TIF) [file pone.0203939.s003.tif]

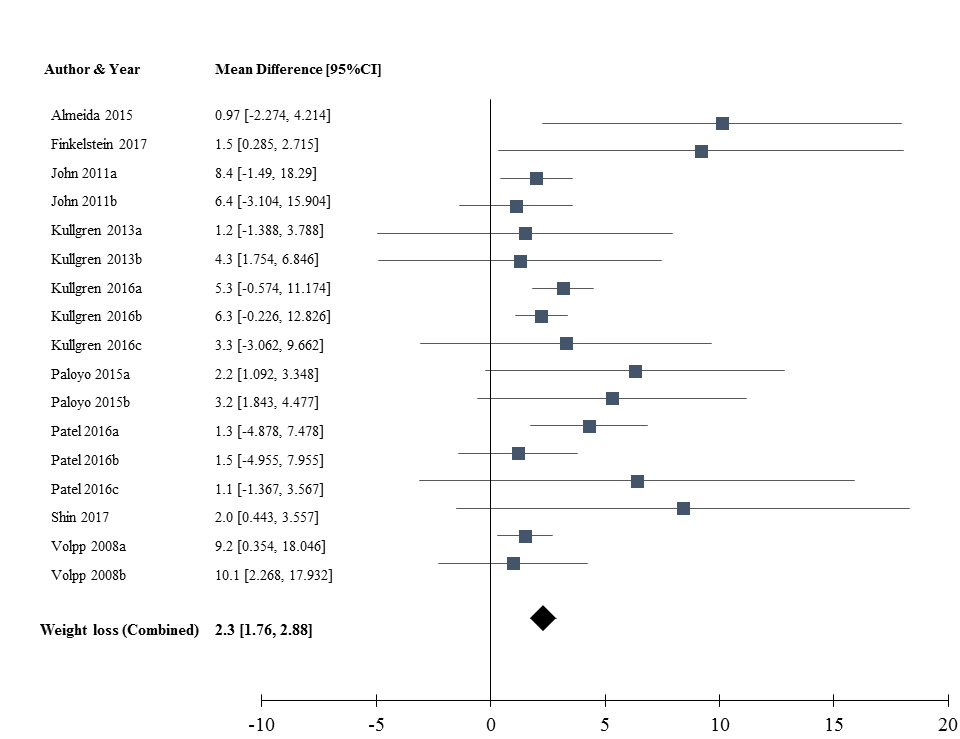

Supplement: S2 Fig — (TIF) [file pone.0203939.s004.tif]
